# *Notes from the Field*: Lead Contamination of Opium — Iran, 2016

**DOI:** 10.15585/mmwr.mm665152a4

**Published:** 2018-01-05

**Authors:** Nasim Zamani, Hossein Hassanian-Moghaddam

**Affiliations:** ^1^Department of Clinical Toxicology, Loghman Hakim Hospital, School of Medicine, Shahid Beheshti University of Medical Sciences, Tehran, Iran; ^2^Excellence Center of Clinical Toxicology, Iranian Ministry of Health, Tehran, Iran.

On February 14, 2016, a patient with known addiction to oral opium and no occupational or other lead exposure was admitted to Loghman-Hakim Hospital and Poison Center (LHHPC) in Tehran, Iran, with abdominal pain, anemia, constipation, and a blood lead level (BLL) of 137 *μ*g/dL (normal = <10 *μ*g/dL). Over the next 8 months, approximately 3,000 oral opium users were evaluated at LHHPC, and found to have elevated BLLs (range = 47–1,124 *μ*g/dL). During February–November 2016, 14 drug couriers who acknowledged transporting illicit substances across international borders in their gastrointestinal tracts (*1*) (“body packers”) were evaluated at LHHPC to determine the lead content of the drugs they were carrying. Abdominopelvic computerized tomography scans were performed on all 14 persons. Four scans demonstrated varying amounts of amorphous radiodense material suggestive of lead; these were the only packs that contained opium. Packs carried by the other 10 couriers contained heroin (two persons), methamphetamine (five), and both heroin and methamphetamine (three). During the evaluation, the couriers were awake, with normal vital signs and physical findings; their BLLs ranged from 2 to 17 *μ*g/dL. They reported having ingested 130, 300, 700, and 1000 g of opium (5–50 packs each) in 20-g to 250-g packs. The packs were expelled intact; a pooled sample of the contents was sent to the chemistry laboratory of Shahid Beheshti University of Medical Sciences in Tehran, where the lead content was found to be 3,553 ppm (equivalent to 3.55 mg/g) by atomic absorption. The study was approved by the Shahid Beheshti University of Medical Sciences Institutional Review Board.

According to the World Health Organization, tolerable weekly intake of lead is 25 *μ*g/kg body weight (*2*) (approximately 0.0018 g per week for a 70-kg [154-lb] adult). The amount of opium consumed by opium users varies widely; published estimates range from 0.6 g/day (*3*) to >100 g/day (*4*). A recent U.S. study of a cluster of heavy metal poisoning among Ayurvedic medication users found BLLs >10 *μ*g/dL in 40% of 115 persons tested and >25 *μ*g/dL in 30% (*5*); the calculated average amount of lead consumed by persons with BLLs >10 *μ*g/dL was 0.03 g/day. If, as in this analysis, contaminated opium contains 3.55 mg lead per gram, a user consuming 10 g of opium per day could be ingesting approximately 0.036 g of lead per day, approximately 20% more than that consumed by the Ayurvedic medicine users who experienced lead toxicity. The rate of absorption of lead from the gastrointestinal tract is variable*; however, the high levels of lead that might be ingested through opium use have the potential to cause substantial lead toxicity, as is currently being reported in Iran (*4*).

Opium is an important cause of lead poisoning in countries with a high prevalence of opium addiction (*4*,*6*), and lead-contaminated opium has previously been reported in Iran (*4*). However, the concentrations of lead in samples obtained by police in 2006 were substantially lower than that found in this analysis (*4*). The reason for high levels of lead in opium seized in Iran has not been determined; however, it is suspected to result from either deliberate adulteration by distributors to make the drug heavier so they can realize more profit or an unintentional addition during the preparation process (*4*). Iran is one of the main pathways for opium trafficking from Afghanistan to the rest of the world. Although opium production in Afghanistan declined by 48% in 2015 (*7*), Afghanistan still accounted for two thirds of the global fields of illicit opium poppy production, and it has been estimated that the 2015 decline will not affect global heroin markets. Although most opiate trafficking to the United States is through South America and Mexico (*7*), some Afghanistan-produced product is supplied to U.S. markets through African countries.

It is not known whether lead is added to other products reportedly transported by drug couriers, including cocaine, heroin, marijuana, hashish, amphetamines, and 3,4-methylenedioxymethamphetamine (“ecstasy”). Clinicians should be aware that persons using opium products that appear to have been smuggled through Iran could be at risk for lead poisoning.

## Footnotes

* Safety evaluation of certain food additives and contaminants. World Health Organization; 2011. http://apps.who.int/food-additives-contaminants-jecfa-database/document.aspx?docID=9003.
